# Public leadership for gender equality: A framework and capacity development approach for gender transformative policy change

**DOI:** 10.1016/j.eclinm.2022.101798

**Published:** 2022-12-26

**Authors:** Alex Munive, Jenn Donville, Gary L. Darmstadt

**Affiliations:** Department of Pediatrics, Global Center for Gender Equality, Stanford University School of Medicine, Stanford, CA 94305, USA

**Keywords:** Public leadership, Feminist leadership, Transformative leadership, Gender equality, Public value, Common good

## Abstract

Public leadership is essential in social change, and pivotal in transforming social and institutional norms related to gender inequality, going well beyond equal representation. It must embrace the potential for all public health leaders, of all genders, to become agents of change who challenge gender injustices and institutionalise gender transformative policies and programmes in public health. To support officials, initially in Ethiopia, and catalyse transformative change, we created a new framework and capacity development approach – Public Leadership for Gender Equality (PL4GE) – which can be customised to respond to each country's context. Drawing from three areas of leadership thought – public, transformative, and feminist leadership – PL4GE takes a public values approach in positioning gender equality as a human right, a common good, and a means to improve health outcomes. PL4GE promotes six key leadership practices – defining purpose and articulating vision, co-creating public value, empowering people, fostering strategic partnerships, navigating power, and embodying personal commitment – and guides public leaders through a capacity development journey of change, facilitating them to identify and activate opportunities for gender transformative change in their work and in turn, more broadly within the public whom they serve.

**Funding:**

10.13039/100000865Bill & Melinda Gates Foundation INV-002664 to 10.13039/100005492Stanford University.

## Introduction – Why public leadership matters for gender equality and health

Public office holders play a critical role in advancing an intentional agenda for gender equality.^1^^,^^2^ They can inspire and enable institutions to take action for social justice and gender equality,^3^ or they can stifle progress, either by design or through inaction.^4^ Many United Nations (UN) agencies and development partners have invested heavily in programmes aiming to increase the presence and voice of women in public life. Given the enormous gender gap in representation, including in the global health sector where women have what is described as an ‘inverted career pyramid’, these efforts are undoubtedly necessary.^5^, ^6^, ^7^, ^8^ However, truly sustainable progress more broadly across sectors requires leaders in the public sphere, both male and female, who understand and embrace gender equality and are equipped to challenge gender injustices and institutionalise gender transformative policies.^9^, ^10^, ^11^

Formal learning opportunities that prepare the most senior public officials for public leadership are limited,^12^ yet there are even fewer resources that can provide guidance for high-level governmental officials on leadership approaches that promote gender equality.^13^ Many leadership development programmes focus on developing individual skills: an approach which ignores over 50 years of studies which have emphasised that leadership is a process of complex interactions between a leader and the context – societal, organisational, and interpersonal – in which they operate.^14^ For example, gender-specific leadership models focus mainly on achieving greater gender balance within organisations rather than taking a systemic approach. The result may be more female leaders working within contexts in which gender bias and discrimination remain institutionalized and hold back the advancement of equality.^15^ In turn, leadership models for the public sector miss the critical role that gender inequality and gender-related social norms play in the choices leaders make and in their overall approach to leadership. Far too often, public sector leadership models avoid exploring whether and how leadership can contribute to gender equality as a common good or in the public sphere.

In order to address these identified gaps and to support high-level public officials in catalysing transformative change, we suggest a new framework and capacity development approach: Public Leadership for Gender Equality (PL4GE). PL4GE proposes a public values approach to gender equality, achieved through leadership competencies and practices that are drawn from three key areas of leadership thought: public value/leadership, transformative leadership, and feminist leadership (Table 1).^16^^,^^17^ Key elements from each of these leadership schools of thought are brought together to create a vision of leadership in which public leaders articulate inclusion and equality in their work and advance gender equality and health as a public value and a common good. In the following sections we define and describe the tenants of PL4GE and explain how they are connected both as concepts but more importantly through leadership practices.

**Table 1:** Key tenets of public leadership, transformative leadership, and feminist leadership which inform the Public Leadership for Gender Equality framework.

	Public leadership	Transformative leadership^16^	Feminist leadership
Starting point	Acknowledges the role of leaders in the creation of public value in the public sphere, similar to how private organisations are concerned with the creation of private value in the market	Acknowledges disparities of power and wealth and how these impinge on the ability of organisations and leaders to achieve their goals, and often discriminate against individuals within these organisations	Recognises that power dynamics and structures of oppression (explicit and implicit) are complex and intersecting along different markers of identity (gender, race, sexuality, ability, location, etc)
Foundation	Understands public value as a common good and the critical role leaders play in the public sphere	Critiques and acknowledges the possibility of change for equality and social justice	Disruptive of the status quo while promoting a change agenda for gender equality and intersectionality
Emphasis	Public sphere and public value/public policies and processes	Social conditions, equity and social change	Gender justice, horizontal leadership, decolonization, amplifying the voices and experiences of individuals and communities who have been historically excluded
Processes	Promotes boundary-crossing and collaborative work Policy and formal norm creation	Reflective, self-awareness and deconstruction of concepts and norms that are the root causes of inequality	Raises critical consciousness, challenges patriarchy, and creates spaces of inclusion and diversity by amplifying the voices and experiences of excluded people
Key values	Public Interest, democracy, citizens as co-creators of what is good for and valued by the public	Liberation, emancipation, democracy, equity, justice	Disruption, care, empathy, well-being, joy, collaboration, and security
Goals	Common good	Societal transformation	Equality and gender equality

## How was the PL4GE framework developed?

To start the PL4GE framework development process, we engaged in a dialogue with prominent former ministers known for promoting gender equality in the public sector, from Martha Karua in Kenya to Geraldine Fraser-Moleketi in South Africa. The objective of these dialogues was to center the inquiry around real challenges and opportunities faced by senior leaders when promoting gender equality in the public sphere.

In parallel, we conducted a mapping of leadership models that could inform this framework. Based on these two processes, three schools of thought were identified for further exploration: public leadership, transformative leadership, and feminist leadership.

We then conducted a literature review as outlined in Panel 1. The review revealed that many traditional competency-building leadership programmes lack a guiding gender intentional conceptual framework, and often, programmes do not respond to the specific national context or specific situation of leaders. Therefore, the PL4GE framework was developed to reflect the elements of a scalable and gender transformative model, but one whose content provides the flexibility to be not just adapted, but deeply contextualised to a variety of countries, regions and sectors. The goal was to design a capacity building programme that supports public leaders to drive forward a locally relevant and impactful gender transformative sector-specific agenda.

Panel 1 **Search strategy and selection criteria**

References for this Review were identified through searches of Google Scholar with the search terms: “gender equality”, “gender transformative”, “transformative leadership”, “transformational leadership”, “feminist leadership”, “public leadership”, “public value”, “ministerial leadership”, and “health leadership”. Given the focus on the public sphere, each search term was run in conjunction with the search terms: “government”, “ministries”, and “public policy”. A second phase of snowballing literature searching was conducted based on the bibliographies of the peer-reviewed articles found in phase one. The search also targeted ‘grey’ literature from UN agencies, governments, international non-governmental organisations and feminist organisations. Articles and documents were also identified through searches of the authors' own files. The search criteria primarily focused on the last 10–15 years, though key literature identified from before was also included. *Only papers published in English were reviewed. The final reference list was generated on the basis of originality and relevance to the broad scope of this Review.*

To support this balance between the design of a gender-integrated model and a high level of contextualisation, a two-tiered structure was created: an advisory and stakeholder group. The advisory group was hosted by Stanford University during 2019–2022 and was composed of specialists in gender, health, leadership and capacity building programmes. The first country for which the programme was contextualised and delivered (in October 2022) was Ethiopia, and there, the development team worked with a stakeholder group of actors that included the Ministry of Women and Social Affairs, UN Women, women's rights organisations, and sectoral ministries. This group was a sounding board and provided valuable input in the development of the PL4GE framework. We also conducted a targeted literature review of prior gender equality programmes in Ethiopia, and developed a case study exploring the integration of considerations of gender equality into the development of Ethiopia's Health Extension Program (HEP).

## Key tenets of public leadership for gender equality

### Overview

Although research on the interconnection between public leadership and gender equality is growing, little analysis has been carried out on public leadership and gender equality theories, or on agreement around competencies and definitions. To help illustrate the connections between public leadership, transformative leadership, and feminist leadership, and set the overall conceptual understanding, we define and unpack the gender equality dimensions of each school of thought, and explain how they are connected both conceptually and through specific leadership practices and, when blended, lead to a unique, synthetic approach to promotion of gender transformative public policies.

These three theories of leadership complement each other to create a more holistic approach that addresses the complex demands of effective public leadership for gender equality. Whereas public leadership theory focuses on the relationship of leaders with, and their obligations to, the public they serve, it does not as a theory articulate a set of embedded values.^17^ Nor does public leadership theory address power relations, and how these are affected by gender and other intersecting social characteristics.^18^^,^^19^ Transformative leadership theory satisfies this requirement of PL4GE by articulating a specific agenda for social justice that emphasises the achievement of equity, democracy and liberation for society. Both transformative leadership theory and feminist leadership theory go a step further by laying out an approach to leadership that not only seeks to achieve social justice as an end goal for public leadership, but proposes that employing a social justice approach to the structures and processes of leadership is equally important. However, whereas transformative leadership theory does not explicitly apply a gendered lens to the role and practice of leadership in the achievement of change, feminist leadership theory articulates this social justice agenda by emphasising the achievement specifically of gender justice and interrogation of patriarchal systems of knowledge, governance and power.

### Public leadership, gender equality and the creation of public value

Leadership in the public sphere is primarily concerned with the creation of public value, which can be understood as the process of diverse actors formulating and/or enacting norms and actions which aim to achieve publicly agreed or sought goals for society.^18^ The main purpose of a good government is to create and sustain public value.^17^^,^^20^ The term “public value” can be understood in two distinct and yet interconnected ways. First, public value and the creation of public value can refer to those goods and services that serve the common good or are beneficial not only to specific individuals but also for the public in general. For example, consider the public value generated through the creation of health, education and criminal justice systems.^21^ Second, public values can refer to those sets of principles or norms which shape the structure of society and our ways of living together; for example the rights, obligations, and protections to which we all (generally) agree.^22^ Whether generated by government, development partners, civil society or the private sector, public value goals (i.e., programmes, policies, systems or services) should be informed and defined by the public values which are commonly held by members of society. For example, where social justice, children's wellbeing and gender equality are held as public values within a society, the health system should reflect those values in order to be recognised as legitimately serving the common good, or in other words: having public value. In this context, public leaders for gender equality aspire for gender equality to be seen both as one of the most critical public values and as a goal and outcome that contributes to the common good.

Public leaders have a role in promoting gender equality as a publicly held value or principle, and in co-creating a vision of gender equality as a common good that benefits everyone. Promoting this understanding is key to energising change: society as a whole must uphold the principle of gender equality as a core value and people must understand that everyone benefits from gender equality, not just girls and women. The co-creation of public value is achieved through ‘boundary-crossing collaborative work’ that can occur through people, processes and structures.^23^ The PL4GE framework draws from this integrative approach, which sees public leadership as ‘the process through which multiple actors with multiple interests create a common vision of, and work together to create, public value’.^23^ This does not negate the valuable role that individual leaders play, but rather recognises the importance of collaborative interactional and relational processes undergirded by principles of equality in the co-creation of public value.^24^

Public leadership theory is primarily concerned with the relationship of leaders to the public they serve, and their obligation to align with and deliver on the values and needs of their specific constituency. However, transformative and feminist leadership approaches articulate a specific agenda for that leadership, and by doing so they advance a specific set of values, including social justice, equity and democracy.

### Transformative leadership

Leadership theories conceptualised as processes of transformation have dominated research within the leadership domain and provided valuable insights to the field of public leadership and the development of the PL4GE framework. Transformative leadership is values-based and has a purposeful agenda for change: it seeks to address objectives of equality, inclusion and social justice.^16^ Transformative leadership is, at its core, a participatory process of creative collaboration and transformation for individual and collective good.^25^ This approach requires the leader to have a clear understanding of the values and beliefs that underpin their own identity, be willing to take a stand and be courageous, and live with the tensions often associated with promoting social change. Transformative leadership emphasises that the goals for change and the processes through which they are achieved need to be connected creatively and in a cohesive way.^26^ The PL4GE draws from this understanding and emphasises leadership that is self-reflective and courageous, and has the specific ambition to create greater social justice. However, most of the literature on transformative leadership does not elaborate how gender is related to leadership practices or why gender bias and discrimination are necessary to address in order to achieve broader social equity and justice. For this reason, feminism and feminist critiques of leadership can provide further insight on how change must also be rooted in addressing gender inequality and patriarchal power structures in order to be transformative.

### Feminist leadership

There is a strong coherence between feminist leadership principles and transformative leadership principles: feminist leadership can be understood as a type of transformative leadership.^27^ While feminist leadership also focuses on the achievement of social justice, it narrows the focus of leadership even further to emphasise justice related to gender equality and the systemic oppression of women and people of other marginalised genders.^28^ The starting point for many feminist leaders is an in-depth exploration of individuals but also organisations on issues of power and privilege. Like transformative leadership, feminist leadership is self-reflective and relational: it creates change through collaboration, empowerment, and consensus-building.^27^ However, feminist leadership also demands that systems of power are interrogated, and prioritises horizontal leadership rather than hierarchical or vertical leadership traditionally characterised by patriarchal and colonial systems of power.^29^ It therefore sees the process of leadership as being equally important as the outcome of leadership. Feminist leadership theories reflect on the fundamental questions of who leads and how, and who has the opportunity to co-create public value and define public values?

## Leading change: From theory to practice

Informed by these three schools of thought, we sought to elicit the connections among them and particularly how they work together to build a coherent framework for leadership and gender equality. We first propose a definition of PL4GE which draws from the key attributes of each theory, and assemble a set of behaviours that leaders in the public space can employ to further an agenda which promotes gender equality as a publicly held value and recognises gender equality as a common good.

Leaders who embody PL4GE are those who are fundamentally guided by principles of social justice and gender equality, which they promote as a public value and recognise as a common good. Such leaders work across horizontal and vertical networks to gain a deep understanding of inequality and to co-create ambitious and feasible solutions. They interrogate and navigate traditional power structures – including as they relate to their own role – to dismantle and redesign structures that serve as barriers to gender justice.

The practical, behavioural implications of embracing PL4GE for high-level governmental officials are outlined below. We have grouped these core activities around six key leadership practices: Purpose, Public Value Co-creation, People, Partnerships, Power, and Personal Commitment (Table 2).

**Table 2:** Key leadership practices and their components for Public Leadership for Gender Equality (PL4GE).

PL4GE practice	Brief description	Components	Practices - examples
Purpose	A shared, long-term vision for gender equality in a sector.	Inspire the creation of a shared vision and a sense of purpose Communicate the purpose and vision Advance long-term sector-specific gender equality outcomes	Articulate the level of aspiration for gender (gender aware or gender transformative) for a particular health policy.
Public value co-creation	Build broad commitment for gender equality as a public value and common good.	Build commitment for gender equality Drive forward game-changing initiatives for gender equality Ensure the realisation of results	Promote gender equality as a fundamental element in health policies and public communications
People	Lead an organisational culture that values diversity and empowers people.	Catalyse organisational culture change Value diversity and inclusion Promote women's leadership at all levels	Promote gender equality as a fundamental element in health policies and public communications
Partnerships	Nurture and employ diverse partnerships that build collaboration for gender equality.	Nurture cross-sectoral relationships Enable participation and cooperation Use interdisciplinary thinking	Build and sustain relationships with other actors in the government and break down silos, particularly by embracing cross-sectoral collaborations that promote shared ownership of gender equality
Power	Understand, navigate, and redistribute power to drive social change.	Be accountable for how power is expressed, distributed and acted upon Navigate the political environment of gender equality	Respond to different forms of resistance to gender equality amongst power-holders
Personal commitment	Vocalise and demonstrate a personal commitment to gender equality.	Exhibit self-awareness Reject discrimination Be vocal about your personal commitment to gender equality as a public value	Use personal behaviour to shape organisational culture Confront and challenge in the workplace, both direct and indirect forms of discrimination, gender inequality, bullying, and sexual harassment

These leadership practices are each distinct but all interconnected, and should be employed in concert, each being an essential component for the achievement of gender equality within the public sphere. However, as with all approaches to the achievement of gender equality, the specific actions and emphasis taken by each individual leader must respond to the social and political context within which they are operating.

### Purpose

Defining and articulating a clear purpose is critical for public leaders who aim to promote gender equality. Social change is a long-term proposition which requires leadership to identify and drive goals that reach far beyond their current term limits. It requires public leadership that is visionary and inspires a sense of shared purpose across and between sectors and stakeholders, and most importantly, amongst the public to which leaders are accountable. Public leaders need **to articulate and communicate purpose and vision of social transformation** in a clear way.^18^ They encourage and motivate others by envisioning a clear, **compelling and achievable** future, articulating how to reach it and setting high standards.

An essential step—and where visionary leadership takes shape—is in the development of transformative goals. Gender-transformative goals aim at catalysing change by examining, questioning, and changing rigid gender norms and imbalances of power. This requires setting ambitious but achievable goals. Ambitious goals can lead to a higher level of performance than can easy-to-achieve or general goals.^30^ Goals should be aspirational to motivate action, attract funding, and help educate the public and stakeholders.^31^ This entails conceptualising leadership as a process of engaging, rallying and motivating people around a specific vision to work collectively towards a shared purpose. Influential public leaders articulate vision not only for their sector but can connect their vision with the country's broader development agenda. A strength of public leadership is its focus on building cross-sector networks to solve complex problems by leveraging shared visions and goals.

### Power

Equality is about how power is distributed and used, and who is privileged by or excluded from the benefits of power. Leaders are accountable for how that power is expressed. An effective leader recognises how to empower others, how to navigate structures of power to make them more representative and just, and how to harness collective power. Therefore, a public leader for gender equality **acknowledges and grapples with power** openly, in part from understanding the different manifestations of power and how to navigate the gender dynamics of power within government.^32^ This requires leaders to **understand and analyse power structures**. They should also urge their teams to **challenge power**, question assumptions, and look at old problems in new ways, such as seeing power as productive when deployed collectively and for the common good. A key role of public leaders is to harness the powers and rally the resources of the state, development partners, the market and civil society behind a common purpose and strategic priorities, in the pursuit of public value goals.^21^^,^^33^

### People

People are the central drivers of change and therefore leaders must empower and engage individuals in a way that reflects and promotes the social change they are seeking more broadly. Gender equality needs effective leadership from women, but also from their male and gender diverse colleagues and from a **wide diversity of individuals and stakeholders** throughout government and partnerships. When leaders make space and actively demonstrate support for diversity, inclusion and empowerment, they galvanise the people around them for social change. Public leaders exert relations-oriented leadership, reflect concern for their constituency's welfare and a desire to foster good interpersonal relations among organisational members.^34^ Public leaders for gender equality also treat team members as equals, appreciating their work and creating opportunities to involve them meaningfully in decision-making processes.^35^ From a gender perspective, this means building equitable relationships and **purposefully fostering the potential of a diversity of people**. For leaders to effectively embrace a gender equality agenda and be successful, they need to recognise that the decision-making process is just as important as achieving organisational goals.

### Public value co-creation

There is a growing recognition that effective public leadership is networked, based on values, and has more impact when utilising transformative and integrative principles rather than transactional practices.^23^^,^^36^ Public leaders’ key role within the co-creation process is to be guarantors for public value by promoting **inclusive platforms for deliberation and collaboration**, informed by evidence and democratic values. In order to be sustained and embedded in systems and society, gender equality must be viewed as a public value – as something to which everyone has a right, and which benefits all. Leading for gender equality means driving initiatives and amplifying public movements that recognise it as a fundamental human right, and critical for the wellbeing and development of all.

### Partnerships

Inequality is complex and systemic, reaching across sectors, throughout government, and into communities. Leaders seeking social justice and equality need to **foster authentic participation and strategic partnerships** with colleagues and those who represent diverse stakeholders, **particularly women and girls and gender diverse individuals**. In most democratic countries, there is a recognition that public value is not only created by the government but also by the public, private, voluntary, and informal community sectors.^37^^,^^38^ This is particularly critical for achieving gender equality. Effective public leaders for gender equality seek to gain a common agreement and be accountable for the public value of gender equality. They seek to **obtain authority and legitimacy where there is often multiple and perhaps conflicting understandings** of the importance of gender equality and how to achieve change. In addition, partnerships are necessary for the generation of collaboration and the co-creation of public value. Through nurtured partnerships, public leaders for gender equality can motivate different actors and incorporate various components to innovate new approaches for the country's development and achievement of social justice.^23^ Leaders must be able to identify and develop partnerships that result in a distributed and networked way to co-produce gender equality as a public value.^37^

### Personal commitment

From a feminist perspective, the division between private and public spheres is seen as artificial.^38^ For feminists, the personal is political and vice versa.^29^ This means that personal change and societal change are intrinsically connected.^35^ The implications of this principle for leadership are far-reaching: leaders must not only **demonstrate or ‘model’ a personal commitment** inside but also outside the organization. A critical trait of public leaders for gender equality is to embody the change they are striving to see in the world. Scholars refer to this as an idealised influence, which means modeling behaviours and practices that motivate constituents and teams to emulate them.^39^ The voice and behaviour of leaders is always amplified – whether through formal or informal channels. When leaders understand and address their own biases and reject discrimination, and actively listen and invite diverse perspectives, they are modeling behaviour for others. When leaders are vocal about their support for equality as an intrinsic right, they are building the case for gender equality as a public value.

What does leading for gender equality in the public health sector look like in practice? Effective application of the theory, principles and practices of PL4GE requires contextualisation. A targeted retrospective review of the case of Ethiopia's Health Extension Program (HEP) offers some insights in terms of the challenges and opportunities faced by leaders while pushing forward public policies and transformative agendas (Panel 2).^40^, ^41^, ^42^, ^43^, ^44^, ^45^, ^46^, ^47^, ^48^, ^49^ In Table 3,^46^^,^^48^^,^^50^, ^51^, ^52^, ^53^, ^54^, ^55^ we identify examples which illustrate how the six key leadership practices outlined above were operationalised, to varying degrees, within the HEP in Ethiopia. The HEP is not being presented as an example, in its totality, of a gender transformative initiative nor was it the inspiration for the design of the PL4GE programme; rather, it serves to illuminate how the six practices which are core to PL4GE can be applied to analyse leadership in a health systems context. The HEP case study was developed as a learning asset and resource to engage and discuss gender equality and leadership aspects with Ethiopian high-level government officials. Development of case studies for other settings will similarly help leaders to envision PL4GE in their context.

Panel 2 **The Health Extension Program (HEP) in Ethiopia**

Notwithstanding its regional disparities, Ethiopia's progress towards meeting the health-related Millennium Development Goals was notable. In just under 20 years, Ethiopia achieved significant strides in raising health-related outcomes for women, including reductions in the maternal mortality ratio by more than 50%^40^ and in unmet need for contraception by 46%,^41^ and a 15% increase in women's decision-making related to health.^42^ Ethiopia's approach has been seen as an innovative model for community-based public health around the world.^43^ Within the programme, certain practices of Public Leadership for Gender Equality (PL4GE) appear and illustrate what application of PL4GE can look like in practice.

In 2003, the Ethiopian government launched an ambitious and innovative approach to public health care that represented a sharp turn from convention. In response to extremely poor health outcomes, especially amongst women and children, Ethiopian leadership proposed and implemented the HEP, which not only responded to the practical challenges of health systems management in rural Ethiopia, but fundamentally shifted the philosophy of public health care from a top-down and curative approach, to one that promoted community-based knowledge and prevention. The HEP specifically aimed to improve the health outcomes of women and children and recognised the centrality of women's health to the achievement of the MDGs.^44^ This created a sense of purpose for the Programme, yet challenges remained.

A key objective was to ensure ‘ownership and participation’ through the transfer of knowledge and skills – and this transfer occurred largely from women-to-women.^45^ However, while women have been the cornerstone of the HEP, both in terms of outcomes and implementation, challenges have also been identified with the degree to which the work of women Health Extension Workers (HEWs) and Women's Development Groups (WDGs) has been recognised, valued and supported.^46^ Despite the role of HEWs in increasing participation of women in the workforce, studies have found that HEWs have been underpaid relative to comparable public sector positions, most have expressed that they feel overloaded by the required tasks, and WDGs are not compensated at all.^47^

There was also resistance to a shift away from a traditional, curative, medically oriented approach to public healthcare and preventative care.^48^ Through the ruling party's political strategy of prioritising rural interests and strong leadership,^49^ however, the ministers and key leaders in government managed to gather support and achieve some remarkable transformation in the lives of its people, particularly women and girls.

**Table 3:** Public Leadership for Gender Equality (PL4GE) practices in the Health Extension Program (HEP) in Ethiopia.

PL4GE practice	Operationalisation of leadership practice in the HEP in Ethiopia
Purpose	Ethiopia's HEP, focused on and driven by women, has been the cornerstone of the Federal Ministry of Health's sector development plans for nearly 20 years, sustained and championed by successive Ethiopian governments and Ministry leaders. Leadership's vision for the HEP has furthermore evolved to reflect an emergent rights-based approach to gender equality seen across sectors in Ethiopia. Strong, consistent and visionary leadership has been credited for ensuring that ‘all Roads’ led to the HEP – the government was in the drivers' seat, and was willing to reject development partnerships and programmes that didn't align with the principles of ownership and vision of the HEP.^48^^,^^50^
Public value co-creation	A philosophy of co-creation of public value and empowerment has been driven by HEP leadership from the outset, reflected in the Daoist quote at the beginning of the HEP's Draft Implementation Plan in 2005: “Go with the people: Live with them. Learn from them. Love them. Start with what they know. Build with what they have. But of the best leaders, when the job is done, the task accomplished, The people will all say, ‘We have done this ourselves’.”^51^ In addition, the HEP purposefully created synergy among public sector, community and development partners, including local and religious leaders, youth and women's associations, and stakeholders across other sectors such as education and agriculture.^48^
People	From the outset, leadership committed to a considerable investment in women's participation and expertise, and gave individual women authority in controlling information. More recently, leadership has amplified their focus on ensuring women's ownership at all levels of leadership – including national Ministry level and at the health facility leadership level.^52^
Partnerships	A key element of the HEP's implementation has been the collaboration and shared ownership of its message and approach by a wide variety of partners and individuals. In particular, the government led a strong partnership that attracted development partners in implementation, monitoring, evaluation, and research.
Power	The HEP represented a purposeful shift from institutional power to individual power, moving the emphasis away from health professionals and into the community. Leaders recognised that progress would require the redistribution of power – downwards to regional levels to create ownership and accountability. More recently, an emphasis has been placed on greater representation of women in power positions and away from male-dominated decision-making, in part a purposeful response to the criticism that the very reason that women are engaged as Health Extension Workers (HEWs) and Women's Development Groups because of their role as traditional caregivers and the household access that this role provides – serves to reinforce gender norms that limit opportunities for women's advancement: ‘female health workers must operate within the same gender systems that necessitate their appointment in the first place’.^46^ Ministry of Health conducted a National HEP Assessment in 2019. The assessment revealed many shortcomings within the HEP, ranging from low skills, high workload, and motivation of HEWs. In response to the study findings and efforts to optimize the HEP, the Ministry of Health leadership embarked on a process resulting in the HEP Roadmap. This document introduced comprehensive benefits and performance-based incentives packages to health post staff and a career path for HEWs.^53^
Personal commitment	Top leadership was unwavering in their commitment to the HEP, and also tolerated the tension of change when moving through and addressing resistance.^54^ “The government was serious about the benefits of HEP and the way it could revolutionize our health system. In particular, our late Prime Minister Meles Zenawi was very committed to the effort. He really believed in the idea of HEP and in primary health care in general as the centerpiece of our health system. So despite the concerns from partners and stakeholders, we really kept pushing.”^55^

## A journey of change for public leadership for gender equality

A critical element of building capacity as a public leader for gender equality is to move from theory to practice. We have developed a five-step Journey of Change to help leaders and organisations supporting leaders to visualise the steps required and build capacity to become Public Leaders for Gender Equality (Panel 3).^56^, ^57^, ^58^, ^59^ This journey is informed by different pedagogical schools of thought in gender equality training and adult learning to create an approach that serves the specific purpose of PL4GE: to build an understanding of the need to, and capacity of public leaders to, govern for gender equality. The PL4GE approach asserts that achieving change in attitudes, perceptions, and behaviours related to gender equality is not a transition that can be ‘taught,’ but rather must be ‘facilitated’ – and necessarily requires the active participation, motivation, and experience of the ‘learner.’^60^ PL4GE is therefore based on facilitation theory, which suggests that the relationship between participant and facilitator is paramount, rather than focusing on the imparting of knowledge or skills in a traditional ‘teaching’ sense. In addition, the program recognises the complementary importance for leaders to understand and master the nationally available gender integration tools and resources.

Panel 3 **Journey of Change: Moving from theory to action in Public Leadership for Gender Equality (PL4GE)**

PL4GE is designed to enhance male and female high-level government officials’ commitment to, and capacity for, developing public policies and leading initiatives that contribute to increased gender equality. Outcomes will be achieved at three levels:

· Individual level: Leaders understand their role and are prepared to promote and embrace gender equality in their professional lives.
· Relational level: Leaders are committed to, and have the capacity for, collaborating and co-creating increased gender-integrated policies and programs to advance gender equality, partnering with their government colleagues but also other key stakeholders, such as development partners, the private sector, and civil society.
· Systemic level: Leaders can identify, and are committed to, a pathway to realise the systemic institutional and social change required for the achievement of gender equality.

**Applying the 6Ps**

The six leadership characteristics – “the 6 Ps” – outlined in Table 2 are the central focus for action, with specific dimensions of practice emphasised at various stages, opportunities to activate those practices in the programme, and exercises that can help build skills to execute those practices in the future. For example, demonstrating personal commitment is a practice that is fostered from the outset of the programme through self-reflection and challenging biases. Participants foster change in their relations with other people throughout the journey, as they practice valuing diversity and examining culture change. Other practices result from the preparation and skills-building that occur through the journey and are activated towards the end of their journey within the programme or after the programme. For example, we aim for progress to be made in nurturing partnerships across sectors and within different organisations and in articulating a long-term purpose and vision for gender equality to a broader constituency. Where participants already possess these dimensions of practice, they are encouraged to strengthen and share, and where participants are building a new skill or practice, they are supported by the experience of their peers and colleagues.

**Grounding in country context**

The first step in preparing for success aims to situate the capacity development approach within a country's movement towards gender equality. In particular, participants are guided to recognise and learn from the role of leadership in previous government successes in the promotion of gender equality, and to critically engage in building on that momentum. The description of the HEP in Ethiopia, for example, was used in this manner. Wherever possible, existing government gender infrastructure (policies, strategies, tools, markers or indicators) are integrated into programme content. The programme focuses on positioning gender equality as a public value or “principle” that can inform all government actions and policies, and uses concrete examples on how this has been done and can be seen in the participating leaders' country context.

**Making it personal**

PL4GE explores gender and leadership by examining leadership through a gender equality lens. It facilitates a process that aims to help leaders recognise their own experience of gender and the role of power and privilege in their own personal life and work, and within their leadership journey. The implications of discriminatory gender norms are far-reaching and affect all aspects of our lives, starting from childhood and continuing throughout our adult lives.^56^ These unequal social norms inform policies, which can be discriminatory and harmful, and which in turn perpetuate social bias, discrimination and marginalisation.^57^ While we all have implicit gender biases and it is important to acknowledge and be aware of them, the work for gender equality does not stop there. Leaders need to understand that gender biases are reflections of societal views, more generally, about the relative value of women and men and how these ideas inform “what is valued and considered acceptable for men and women. In most societies, norms tend to value and privilege what is male over what is female”.^58^ Finally, in this phase of the journey, attention is given for leaders to understand different types of change that can be achieved in response to gender inequality, recognising transformative change as most effective.^59^

**Taking action**

PL4GE explores the Six Ps using concrete examples that outline the critical nature of each of the practices. Examples are provided that illustrate the value of working with diverse partners, including partnering with women's rights organisations. Leaders are encouraged to identify leadership approaches and the tools that are needed in order to achieve gender-transformative change through government. High-level government officials are presented with different tools, including a gender equality marker which is an accountability tool that examines the degree to which a policy, programme, or institution has integrated gender equality in their work. The tool can help identify gender equality strengths and opportunities within their own ministries by applying a gender equality lens to their own work and plans.

The programme aims to turn insights into action by supporting leaders to identify change goals that are transformative, feasible, and contextualised. It builds on the understanding that effective change for gender equality involves both incremental goals and goals that contribute to long-term visionary changes that are gender transformative. In order for change to be sustained, gender transformative objectives aim at catalysing change by examining, questioning, and changing rigid gender norms and imbalances of power. Four key criteria apply to setting objectives that are visionary and can have long term impacts. Goals need to be aspirational, aligned to national and health development plans, responsive to women and girls, and focused on addressing root causes of gender equality. Finally, during this phase, leaders are encouraged to reflect on their own responsibility for the achievement of social justice and gender equality. They make a commitment to realise this change in their day-to-day work, as well as their long-term aspirations in government/leadership.

During the consolidation and celebration phase of the programme, leaders review how they have built on their own knowledge and experience to recognise their role in the promotion of gender equality as a public value. They make a commitment to each other to provide support and collaboration in their leadership roles towards realising their goals for change.

Therefore, the journey of change is connected to specific learning and reflection moments that are facilitated within a capacity building programme, but also goes beyond it. This journey is built to provide leaders with personal space and time to reflect on their own values and how they can shape their behaviour, learn about gender mainstreaming tools, and work to identify game-changing initiatives for gender equality with which they want to be associated, and more importantly, to plan for how to get the work done. In order to enhance reflection, critical thinking, and envision ambitious but feasible solutions for a particular context, PL4GE proposes investing in research and consultation to specifically respond to each country's national context, not only by developing and using case studies drawn from each country's own track record on gender equality (e.g., HEP in Ethiopia) but also by engaging and documenting personal accounts and reflections from public leaders in particular context and by working directly with the gender equality machinery of a country, including for example the Gender or Women Directorates within the Ministry of Health.

The journey of change includes different steps that, taken together, can lead to a significant impact on leadership, health and gender equality. It is designed as a process where leaders reflect and answer critical questions that will support them in defining the best pathway forward to promote and support gender equality for their specific sector. Moreover, the focus is not only on enhancing leaders’ understanding of their role, enabling them to promote and embrace gender equality in their professional lives, and developing their capacity to collaborate and co-create gender-integrated policies, but ultimately aims for leaders to be able to identify a pathway to realise the systemic institutional and social changes required for the achievement of gender equality. It is also important to note that no two journeys will be the same, so the journey is designed to be individualised, participatory, and iterative, rather than prescriptive.

## Outstanding questions

Those in public positions of leadership today must not only understand and embrace gender equality as a fundamental human right, but must also become equipped to challenge gender injustices and institutionalise gender transformative policies in their own context. This requires both an understanding of theory as well as putting into practice principles of PL4GE. We propose a public values approach to gender equality, achieved through personal and collective reflection and development of leadership competencies and practices that are drawn from three key areas of leadership thought: public value/leadership, transformative leadership, and feminist leadership. For application of this proposed approach, we offer a set of specific leadership practices that can be particularly effective in the promotion of gender equality in the public sphere and the health sector. These include articulating a clear purpose, co-creating public value, empowering people, fostering strategic partnerships, navigating power, and embodying personal commitment. The Six Ps are a useful framework to motivate action and self-reflection amongst leaders, and they provide the flexibility to be contextualised and individualised for each leader in their context and within their particular remit.

The PL4GE framework and journey of capacity development can support public leaders in the health sector, and their colleagues at the helm of other public sectors, to visualise and activate institutional change for gender equality. A key next step is to generate a deeper understanding of how to most effectively translate the framework to serve different global contexts with diverse leadership and gender equality and health challenges, with an aim of establishing a responsive and adaptive model of leadership for gender equality in public health.

### Role of the funding source

The funders of the study had no role in study design, data collection, data analysis, data interpretation, or writing of the report. All authors had full access to the data in the study and the corresponding author had final responsibility to submit the paper for publication.

## Contributors

A.M., J.D. and G.L.D. conceptualised the paper; G.L.D. acquired funding, managed project administration and resources, and provided supervision; A.M. and J.D. conducted the literature review and synthesis, led data visualization and wrote the original draft; all authors contributed to writing review and editing. All authors contributed intellectual content and approved the final draft for publication. All authors had full access to the data in the study and take responsibility for the integrity and accuracy of the content.

## Declaration of interests

The authors declare no conflicts of interest.
